# Supplementary material for: Resolving the full spectrum of human genome variation using Linked-Reads
Source: Genome Res. 2019 Apr;29(4):635–45. doi: 10.1101/gr.234443.118 (PMC6442396; doi:10.1101/gr.234443.118)
Supplement: Supplemental Material [file supp_gr.234443.118_Supplemental_longranger-2.2.zip › longranger-2.2.tar.bz2!/lr2.2-public/lib/stan/cmdstan/stan/doc/api/html/classstan_1_1agrad_1_1vari.html]

Stan: stan::agrad::vari Class Reference


|  |  |
| --- | --- |
|  | Stan  2.5.0  probability, sampling & optimization |


- Main Page
- Related Pages
- Namespaces
- Classes
- Files

- Class List
- Class Index
- Class Hierarchy
- Class Members

All Classes Namespaces Files Functions Variables Typedefs Enumerations Enumerator Friends Macros Pages

- stan
- agrad
- vari

Public Member Functions |
Public Attributes |
Friends |
List of all members

stan::agrad::vari Class Reference

The variable implementation base class.
More...

`#include <vari.hpp>`

Inheritance diagram for stan::agrad::vari:

|  |  |
| --- | --- |
| Public Member Functions | |
|  | vari (const double x) |
|  | Construct a variable implementation from a value. More... |
|  | |
|  | vari (const double x, bool stacked) |
|  | |
| virtual | ~vari () |
|  | Throw an illegal argument exception. More... |
|  | |
| virtual void | init\_dependent () |
|  | Initialize the adjoint for this (dependent) variable to 1. More... |
|  | |
| virtual void | set\_zero\_adjoint () |
|  | Set the adjoint value of this variable to 0. More... |
|  | |
| Public Member Functions inherited from stan::agrad::chainable | |
|  | chainable () |
|  | Construct a chainable object. More... |
|  | |
| virtual | ~chainable () |
|  | Chainables are not destructible and should go on the function call stack or be allocated with operator new. More... |
|  | |
| virtual void | chain () |
|  | Apply the chain rule to this variable based on the variables on which it depends. More... |
|  | |

|  |  |
| --- | --- |
| Public Attributes | |
| const double | val\_ |
|  | The value of this variable. More... |
|  | |
| double | adj\_ |
|  | The adjoint of this variable, which is the partial derivative of this variable with respect to the root variable. More... |
|  | |

|  |  |
| --- | --- |
| Friends | |
| class | var |
|  | |
| std::ostream & | operator<< (std::ostream &os, const vari \*v) |
|  | Insertion operator for vari. More... |
|  | |

|  |  |
| --- | --- |
| Additional Inherited Members | |
| Static Public Member Functions inherited from stan::agrad::chainable | |
| static void \* | operator new (size\_t nbytes) |
|  | Allocate memory from the underlying memory pool. More... |
|  | |
| static void | operator delete (void \*) |
|  | Delete a pointer from the underlying memory pool. More... |
|  | |

## Detailed Description

The variable implementation base class.

A variable implementation is constructed with a constant value. It also stores the adjoint for storing the partial derivative with respect to the root of the derivative tree.

The chain() method applies the chain rule. Concrete extensions of this class will represent base variables or the result of operations such as addition or subtraction. These extended classes will store operand variables and propagate derivative information via an implementation of chain().

Definition at line 28 of file vari.hpp.

## Constructor & Destructor Documentation

|  |  |  |  |  |  |
| --- | --- | --- | --- | --- | --- |
| stan::agrad::vari::vari | ( | const double | *x* | ) |  |

inline

Construct a variable implementation from a value.

The adjoint is initialized to zero.

All constructed variables are added to the stack. Variables should be constructed before variables on which they depend to insure proper partial derivative propagation. During derivative propagation, the chain() method of each variable will be called in the reverse order of construction.

Parameters
:   |  |  |
    | --- | --- |
    | x | Value of the constructed variable. |

Definition at line 57 of file vari.hpp.

|  |  |  |  |
| --- | --- | --- | --- |
| stan::agrad::vari::vari | ( | const double | *x*, |
|  |  | bool | *stacked* |
|  | ) |  |  |

inline

Definition at line 63 of file vari.hpp.

|  |  |  |  |  |
| --- | --- | --- | --- | --- |
| virtual stan::agrad::vari::~vari | ( |  | ) |  |

inlinevirtual

Throw an illegal argument exception.

*Warning*: Destructors should never called for agrad objects.

Exceptions
:   |  |  |
    | --- | --- |
    | Logic | exception always. |

Definition at line 79 of file vari.hpp.

## Member Function Documentation

|  |  |  |  |  |
| --- | --- | --- | --- | --- |
| virtual void stan::agrad::vari::init\_dependent | ( |  | ) |  |

inlinevirtual

Initialize the adjoint for this (dependent) variable to 1.

This operation is applied to the dependent variable before propagating derivatives.

Reimplemented from stan::agrad::chainable.

Definition at line 88 of file vari.hpp.

|  |  |  |  |  |
| --- | --- | --- | --- | --- |
| virtual void stan::agrad::vari::set\_zero\_adjoint | ( |  | ) |  |

inlinevirtual

Set the adjoint value of this variable to 0.

Reimplemented from stan::agrad::chainable.

Definition at line 95 of file vari.hpp.

## Friends And Related Function Documentation

|  |  |  |  |
| --- | --- | --- | --- |
| std::ostream& operator<< | ( | std::ostream & | *os*, |
|  |  | const vari \* | *v* |
|  | ) |  |  |

friend

Insertion operator for vari.

Prints the current value and the adjoint value.

Parameters
:   |  |  |
    | --- | --- |
    | os | [in,out] ostream to modify |
    | v | [in] vari object to print. |

Returns
:   The modified ostream.

Definition at line 108 of file vari.hpp.

friend class var

friend

Definition at line 30 of file vari.hpp.

## Member Data Documentation

double stan::agrad::vari::adj\_

The adjoint of this variable, which is the partial derivative of this variable with respect to the root variable.

Definition at line 43 of file vari.hpp.

const double stan::agrad::vari::val\_

The value of this variable.

Definition at line 37 of file vari.hpp.

---

The documentation for this class was generated from the following file:

- src/stan/agrad/rev/vari.hpp


---

[ Stan Home Page ]

*© 2011–2014,
Stan Development Team.*
